# Complete mitochondrial genome and the phylogenetic position of the snaggletooth shark *Hemipristis elongata* (Carcharhiniformes: Hemigaleidae)

**DOI:** 10.1080/23802359.2016.1197074

**Published:** 2016-07-23

**Authors:** Xiaolin Huang, Junqi Yu, Hao Chen, Xiao Chen, Junjie Wang

**Affiliations:** a Zhejiang Mariculture Research Institute, Wenzhou, Zhejiang, P.R. China;; b Department of Marine Biotechnology, School of Life Science, Wenzhou Medical University, Wenzhou, P.R. China;; c College of marine science, South China Agriculture University, Guangzhou, P.R. China;; d Key Laboratory of Tropical and Subtropical Fishery Resource Application and Cultivation, Ministry of Agriculture, Pearl River Fisheries Research Institute of Chinese Academy of Fishery Sciences, Guangzhou, Guangdong, P.R. China

**Keywords:** *Hemipristis elongata*, Hemigaleidae, mitochondrial genome

## Abstract

In this study, we first present the complete mitochondrial genome of *Hemipristis elongata*, the only member of genus *Hemipristis* in family Hemigaleidae. It is 16,691 bp in length with the typical gene order invertebrates. Its overall base composition is 31.7% A, 24.1% C, 12.9% G and 31.3% T. Two start codons (ATG and GTG) and three stop codons (TAG, AGA and TAA/T) are found in the protein-coding genes. The 22 tRNA genes ranged from 67 bp (tRNA-*Cys*, tRNA-*Ser*2) to 75 bp (tRNA-*Leu*1). The phylogenetic result showed that *H. elongata* was clustered to *Hemigaleus microstoma* and formed Hemigaleidae.

The snaggletooth shark (*Hemipristis elongata*) is the only member of genus *Hemipristis* in family Hemigaleidae. It has a slender body, commonly in habiting in inshore and offshore on the continental and insular shelves of Indo-West Pacific (Compagno 1984; Carpenter et al. 1997). The reproductive modes of *H. elongata* is viviparous, with 2–11 young in a litter after a gestation period of 7–8 months (Last & Stevens 1994). In this study, we determined the complete mitochondrial genome of *H. elongata* for the first time, and constructed the phylogenetic tree using the mitogenomes in Carcharhiniformes.

One specimen of *H. elongata* was captured in Ranong, Thailand and preserved in the Museum of Marine Biology in Wenzhou Medical University with voucher: RN2012122416. The experimental protocol and data analysis methods followed Chen et al. (2014). Including *H. elongata*, twenty-nine species of Carcharhiniformes with the complete mitogenomes available in the GenBank were selected to construct the phylogenetic tree. The Bayesian method was fulfilled with the GTR + I + G model by three partitions of the mitogenomic data:12S and 16S rRNA genes, and the first and second codons of the 12 protein-coding genes (except *ND6* gene).

The complete mitochondrial genome of *H. elongata* is 16,691 bp in length (Genbank Accession Number: KU508621). It contains 13 protein-coding genes, 2 rRNA genes, 22 tRNA genes and a control region. The gene composition, arrangement and transcriptional orientation are identical to most mitogenomes of vertebrates. Its nucleotide base composition is 31.7% A, 24.1% C, 12.9% G and 31.3% T. There were total 23 bp short intergenic spaces located in 12 gene junctions and 33bp overlaps located in 10 gene junctions in the mitogenome. Both 12S rRNA (953 bp) and 16S rRNA (1666 bp) genes were between tRNA-*Phe* and tRNA-*Leu1* genes, separated by tRNA-*Val* gene. All protein-coding genes started with the standard ATG codon except *CO1* gene, which used GTG as the start codon. Except for the *ND6* gene using AGA as the stop codon, the remaining protein-coding genes were terminated by the typical TAG or TAA/T codon. Twenty-two tRNA genes interspersed between the rRNA and protein-coding genes, ranging from 67bp (tRNA-*Cys* and tRNA-*Ser*2) to 75 bp (tRNA-*Leu*1). Except for the tRNA-*Ser2* that replaced the dihydrouridine arm by a simple loop, the remaining tRNAs could fold into a typical clover-leaf secondary structure. The control region was 1063 bp in length, with the rich in A + T (67.1%) and poor in G (12.6%) content.

Six families of Carcharhiniformes were included in the phylogenetic tree. Most nodes of the Bayesian tree were well supported (Figure 1). The relationship of three basal families (Scyliorhinidae, Pseudotriakidae and Triakidae) was clear. *Hemipristis elongata* was clustered to *Hemigaleus microstoma* with high support value (94%), however, this clade (Hemigaleidae) was then clustered to *Galeocerdo cuvier* (Carcharhinidae) with low support value (53%). In addition, Sphyrnidae were inserted between two main clades of (*G. cuvier* + Hemigaleidae) and the remaining Carcharhinidae species, it demonstrated that Carcharhinidae is paraphyletic. Therefore, the relationship of the families (Carcharhinidae, Hemigalidae and Sphyrnidae) in Carcharhiniformes need further studies.

**Figure 1.**
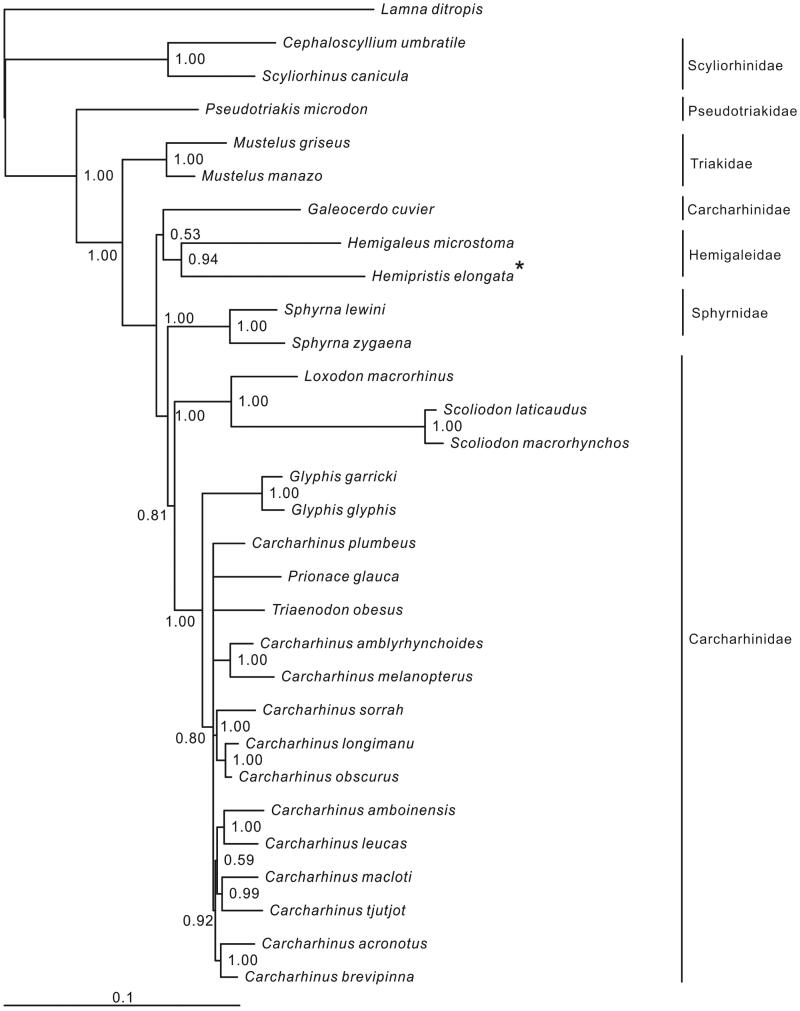
Phylogenetic position of *Hemipristis elongata*. *Lamna ditropis* (KF962053.1) was selected as the out group. The twenty-nine species from the order Carcharhiniformes were: *Carcharhinus acronotus* (NC_024055.1), *C. amblyrhynchoides* (NC_023948.1), *C. amboinensis* (NC_026696.1), *C. brevipinna* (KM244770.1), *C. leucas* (KF646785.1), *C. longimanu* (NC_025520.1), *C. macloti* (NC_024862.1), *C. melanopterus* (NC_024284.1), *C. obscurus* (NC_020611.1), *C. plumbeus* (NC_024596.1), *C. sorrah* (NC_023521.1), *C. tjutjot* (KP091436.1) *Galeocerdo cuvier* (NC_022193.1), *Loxodon macrorhinus* (KT347599), *Prionace glauca* (NC_022819.1), *Scoliodon laticaudus* (KP336547.1), *S. macrorhynchos* (NC_018052.1), *Triaenodon obesus* (KJ748376.1), *Glyphis glyphis* (NC_021768.2),*G. garricki* (KF646786.1), *Mustelus griseus* (NC_023527.1), *M. manazo* (NC_000890.1), *Cephaloscyllium umbratile* (KT003686), *Hemigaleus microstoma* (KT003687), *Hemipristis elongata* (KU508621), *Scyliorhinus canicula* (NC_001950.1), *Pseudotriakis microdon* (NC_022735.1), *Sphyrna lewini* (NC_022679.1), *Sphyrna zygaena* (NC_025778.1).

## References

[CIT0001] Carpenter KE, Krupp F, Jones DA, Zajonz U. 1997 FAO species identification field guide for fishery purposes In: Living marine resources of Kuwait, Eastern Saudi Arabia, Bahrain, Qatar, and the United Arab Emirates. Rome: FAO p. 293.

[CIT0002] Chen X, Ai W, Xiang D, Chen S. 2014 Complete mitochondrial genome of the red stingray *Dasyatis akajei* (Myliobatiformes: Dasyatidae). Mitochondrial DNA. 25:37–38. 23841617 10.3109/19401736.2013.779262

[CIT0003] Compagno LJV. 1984 FAO fisheries synopsis no. 125 In: FAO species catalogue. vol. 4. Sharks of the world. An annotated and illustrated catalogue of shark species known to date. Part 2: Carcharhiniformes. Rome: FAO p. 251–655.

[CIT0004] Last PR, Stevens JD. 1994 Sharks and rays of Australia. Australia: CSIRO p. 513.

